# Practical application of Six Sigma management in analytical biochemistry processes in clinical settings

**DOI:** 10.1002/jcla.23126

**Published:** 2019-11-27

**Authors:** Bingfei Zhou, Yi Wu, Hanlin He, Cunyan Li, Liming Tan, Youde Cao

**Affiliations:** ^1^ Clinical Laboratory of Hunan Provincial People's Hospital The First Affiliated Hospital of Hunan Normal University Changsha China; ^2^ Research Office of Clinical Laboratory, Clinical Translational Medicine Research Institute of Hunan Provincial People's Hospital, The First Affiliated Hospital of Hunan Normal University Changsha China; ^3^ Department of Medical laboratory of Hunan Normal University School of Medicine Changsha China

**Keywords:** quality assurance, quality control, quality goal index, root cause analysis, Six Sigma

## Abstract

**Background:**

Six Sigma methodology with a zero‐defect goal has long been applied in commercial settings and was utilized in this study to assure/improve the quality of various analytes.

**Methods:**

Daily internal quality control (QC) and external quality assessment data were collected and analyzed by calculating the sigma (σ) values for 19 analytes based on the coefficient of variation, bias, and total error allowable. Standardized QC sigma charts were established with these parameters. Quality goal index (QGI) analysis and root cause analysis (RCA) were used to discover potential problems for the analytes.

**Results:**

Five analytes with σ ≥ 6 achieved world‐class performance, and only the Westgard rule (1_3s_) with one control measurement at two QC material levels (N2) per QC event and a run size of 1000 patient samples between QC events (R1000) was needed for QC. In contrast, more control rules (2_2s_/R_4s_/4_1s_) along with high N values and low R values were needed for quality assurance for five analytes with 4 ≤ σ < 6. However, the sigma levels of nine analytes were σ < 4 at one or more QC levels, and a more rigorous QC procedure (1_3s_/2_2s_/R_4s_/4_1s_/8_x_ with N4 and R45) was implemented. The combination of QGI analysis and RCA further revealed inaccuracy or imprecision problems for these analytes with σ < 4 and discovered five aspects of potential causes considered for quality improvement.

**Conclusions:**

Six Sigma methodology is an effective tool for evaluating the performance of biochemical analytes and is conducive to quality assurance and improvement.

## 1 INTRODUCTION

The current clinical study examined the utility of internal quality control (IQC) and external quality control (EQC) for the quality assurance (QA) of biochemical analytes. However, the development of precision medicine has resulted in increased challenges regarding quality control (QC). A system that integrates accurate evaluation, problem‐solving, and process improvement is required, and the Six Sigma management methodology has thus attracted public attention.

The Six Sigma management method was proposed by Bill Smith (an engineer at Motorola), later introduced in China in the late 1990s and started to be applied in hospital management after 1999.^1^ The Six Sigma management model includes five processes, namely define, measure, analyze, improve, and control (DMAIC). In mathematical fields, sigma is the symbol for standard deviation (SD).^2^ Some studies have shown that sigma metrics can be applied to quantitatively evaluate errors or defects in testing projects in clinical laboratories, and the results are quantified as defects per million (DPMs).^3^, ^4^ The Six Sigma metric corresponds to 3.4 DPM opportunities in a clinical process. To date, sigma methodology has mainly been applied in pre‐analytical and analytical processes in clinical laboratories, focusing on the evaluation of biochemical and immunoassay tests.^4^, ^5^, ^6^, ^7^, ^8^, ^9^, ^10^, ^11^

In this study, the performance of 19 analytes was evaluated by calculating sigma values from the coefficient of variation (CV), bias, and total error allowable (TEa). Moreover, appropriate QC procedures were selected for each analyte using the sigma metrics. In addition, quality goal index (QGI) analyses and root cause analysis (RCA) were further performed to identify problems related to the measurement procedures for analytes with a sigma value below 4.

## 2 MATERIALS AND METHODS

### 2.1 Data collection

The internal quality control data required for this study were extracted between January 1 and May 31, 2018, using an AU5800 analyzer (Beckman Coulter) at our clinical biochemical laboratory. The AU5800 analyzer is a modular combination system that includes P1 and P2 analysis modules and a solo ion‐selective electrode (ISE) module. The following 16 analytes were tested using both analysis modules: total protein (TP), albumin (ALB), total bilirubin (TBIL), alanine aminotransferase (ALT), aspartate aminotransferase (AST), alkaline phosphatase (ALP), γ‐glutamyl transpeptidase (γ‐GT), blood urea (BUN), creatinine (CRE), uric acid (UA), glucose (GLU), triglyceride (TG), total cholesterol (TC), creatine kinase (CK), calcium (Ca), and phosphorus (P). In addition, sodium (Na), potassium (K), and chlorine (Cl) were analyzed using the ISE module. The daily QC material Level 1 (LOT: 26411, used at a normal concentration) and Level 2 (LOT: 26412, used at an abnormal concentration) used in this study were purchased from Bio‐Rad Laboratories Inc.

External quality control (EQC) data were collected from external quality assurance schemes of the National Center for Clinical Laboratories (NCCL) of China between 2016 and 2017. According to the requirements of external quality assessment (EQA) for clinical laboratories (GB/T 20470‐2006), the EQA activities were implemented three times per year in the biochemical routine projects conducted in our laboratory. Each EQA activity included the testing of five samples of the 19 analytes, and five bias values were obtained accordingly for every analyte. Thus, 2‐year accumulative bias data were used to calculate the average value, which was used to evaluate the system error in terms of accuracy for every analyte. In addition, it is worth noting that once the nonconformity of an EQA activity (score < 80%) for an analyte was observed, the bias data for the corresponding analyte in the EQA activity would not be included in the analysis.

### 2.2 Construction of the standardized QC sigma charts

The frame of the standardized QC sigma charts was constructed by registering a CLInet account in CLInet (http://www.clinet.com.cn) and inputting parameters such as TEa, bias, and CV in the interface of the Six Sigma management menu. The construction of the standardized QC sigma charts obeyed the concept of previously reported studies.^12^, ^13^ This approach allows a laboratory to obtain an audiovisual and comprehensive view of the performance of all the analytes in a single graph at every control measurement level and with every instrument module.

### 2.3 RCA

The RCA method was performed as previously described.^14^, ^15^ The cause‐effect chart (fishbone diagram) was used as a technical tool for RCA. To date, RCA has been applied to solve problems in the field of medical management.^16^

### 2.4 Statistical analysis

The CV was used to indicate the precision and was calculated with the following formula: $\text{CV}\left(\%\right)=\left[\text{Standard Deviation}\left(\text{SD}\right)/\text{Mean}\right]\times 100$.

The following formula was used to calculate every bias value for each EQA activity: $\text{Bias}\left(\%\right)=\left(|\text{measurement value}-\text{target value}|/\text{target value}\right)\times 100$. The median of the EQA results reported by clinical laboratories that used the same type of instrument and method was used as the target value for every analyte.

The TEa was determined according to the proficiency testing criteria of American Clinical Laboratory Improvement Amendment 88 (CLIA88).

Sigma metrics were calculated with the following formula: $\text{Sigma}=\left(\text{TEa}-\text{Bias}\right)/\text{CV}$.

The QGI was calculated using the formula $\text{QGI}=\text{Bias}/\left(1.5\times \text{CV}\right)$. This index can help determine the main reason why the testing performance of a clinical chemistry project yields a lower sigma level and might aid the selection of the best quality improvement plan.^8^, ^9^, ^17^ A sigma value less than 4 (σ < 4) was used as the benchmark for the QGI analysis of analytes in this study. A QGI value less than 0.8 (QGI < 0.8) indicates that the precision of the corresponding analyte needs to be improved, whereas a value greater than 1.2 (QGI > 1.2) indicates that the accuracy of the analyte needs to be improved. A QGI value between 0.8 and 1.2 (0.8 ≤ QGI ≤ 1.2) indicates that the accuracy and precision of the analyte need to be simultaneously improved.

## 3 RESULTS

### 3.1 Use of sigma metrics for the evaluation of analyte performance

**Figure 1:**
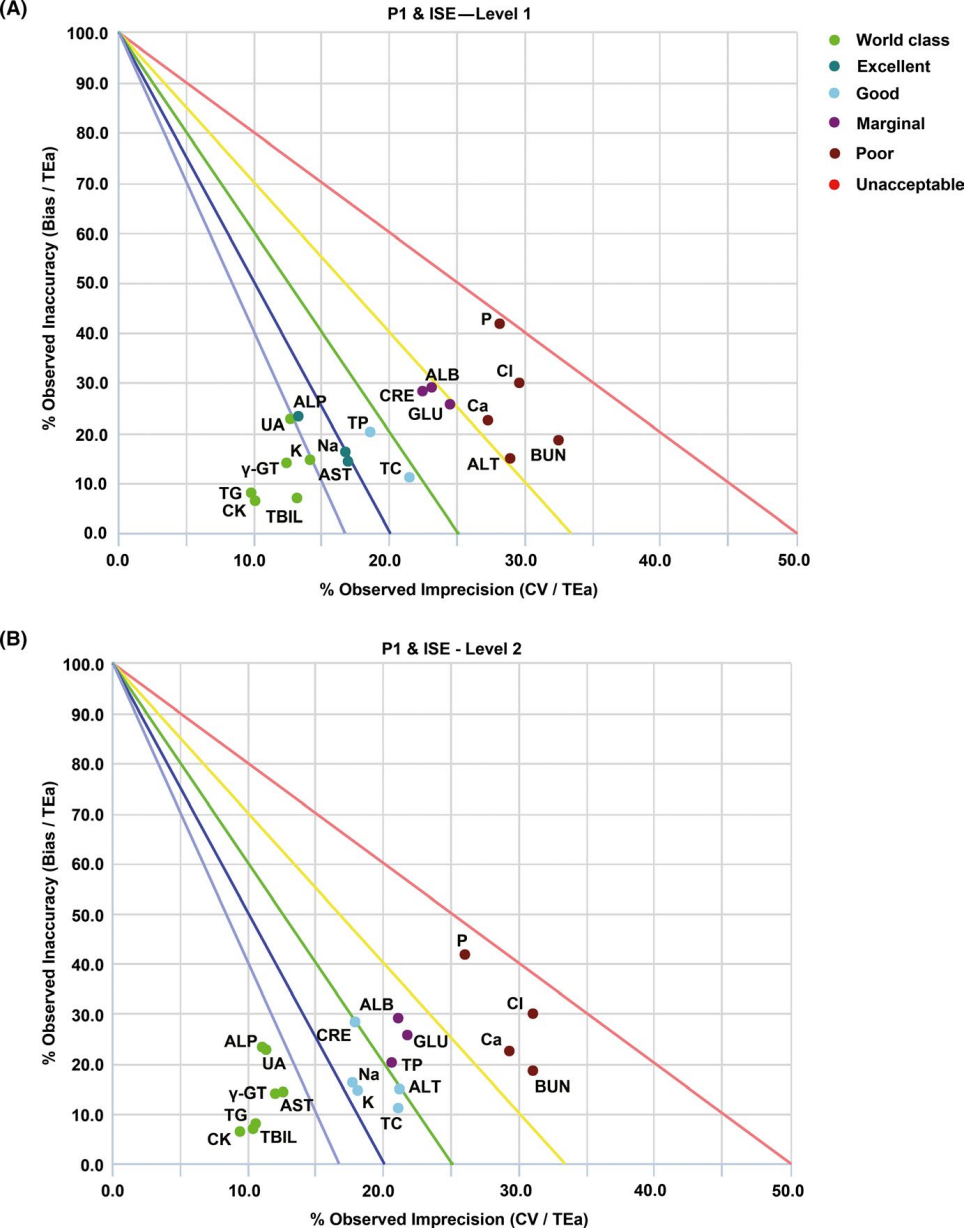
Standardized QC sigma charts for 19 analytes (Levels 1 and 2) analyzed with the P1 and ISE modules of AU5800. (A) QC chart for QC material Level 1. (B) QC chart for QC material Level 2. The slope of the five lines is the negative value of sigma. The circles with different colors represent different sigma grades. The abscissa is the percentage of CV normalized to TEa and shows the imprecision, and the ordinate is the percentage of bias normalized to TEa and shows the inaccuracy

**Figure 2:**
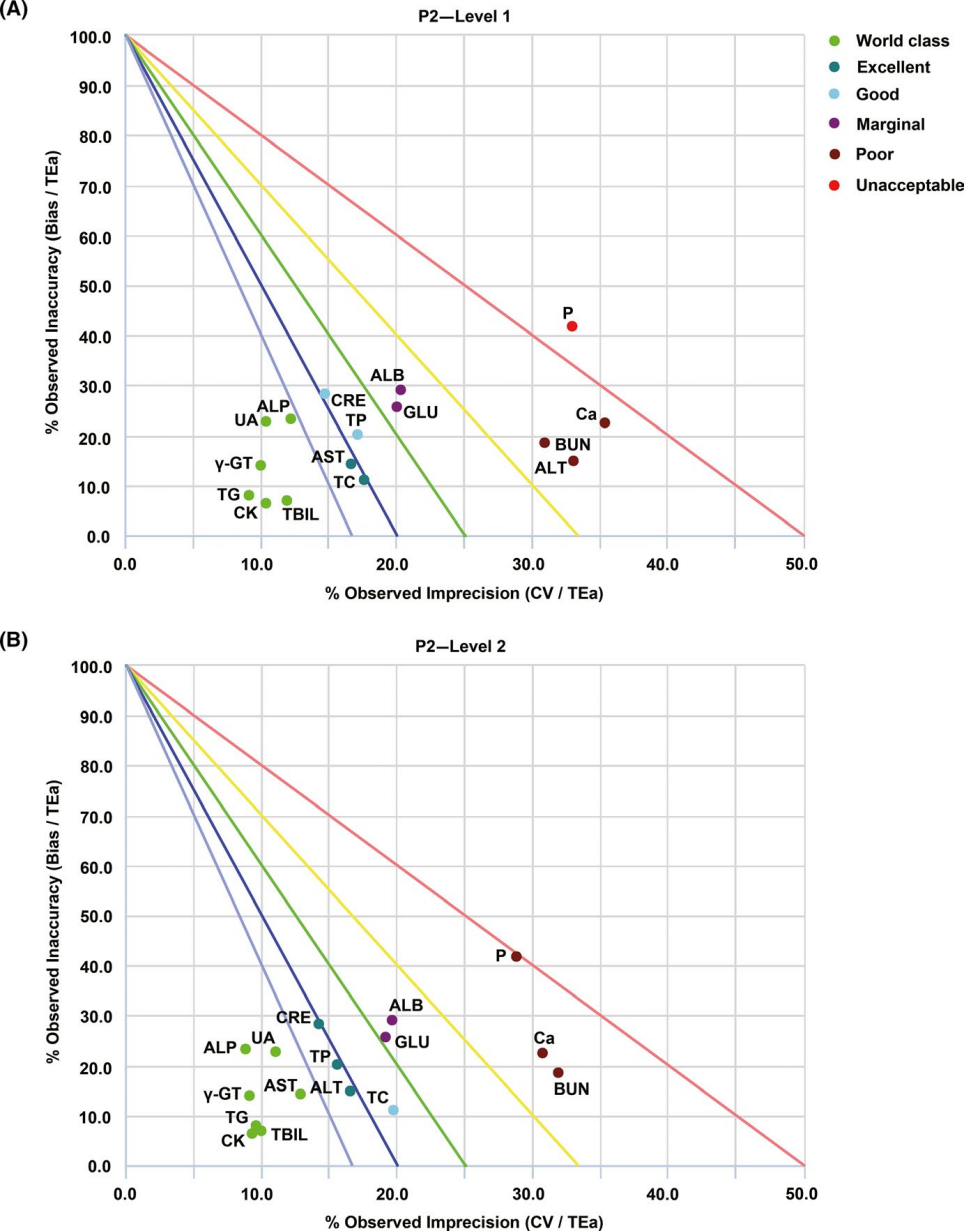
Standardized QC sigma charts for 16 analytes (Levels 1 and 2) analyzed with the P2 module of AU5800. (A) QC chart for QC material Level 1. (B) QC chart for QC material Level 2. The slope of the five lines is the negative value of sigma. The circles with different colors represent different sigma grades. The abscissa is the percentage of CV normalized to TEa and shows the imprecision, and the ordinate is the percentage of bias normalized to TEa and shows the inaccuracy

To understand the performance of the 19 analytes on the AU5800 P1, P2, or ISE modules in our laboratory, the sigma metrics of every analyte at the QC material Levels 1 and 2 were calculated and are summarized in Tables 1 and 2. Furthermore, standardized QC sigma charts were constructed to intuitively evaluate the performance of the analytes at every QC material level and with every module. According to the sigma level, the performance of the analytes was divided into six grades, namely world class (σ ≥ 6), excellent (5 ≤ σ < 6), good (4 ≤ σ < 5), marginal (3 ≤ σ < 4), poor (2 ≤ σ < 3), and unacceptable (σ < 2), as shown in Figures 1 and 2.

**Table 1:** Sigma metrics (Levels 1 and 2) for 19 analytes obtained using the P1 analysis module or ISE module of AU5800 calculated based on the TEa, bias (%), and CV (%, Levels 1 and 2)

Analytes	TEa (%, CLIA)	Bias (%)	CV (%)		Sigma	
			Level 1	Level 2	Level 1	Level 2
CK	30.00	1.92	3.02	2.82	9.30	9.96
TG	25.00	2.00	2.44	2.64	9.43	8.71
TBIL	20.00	1.39	2.64	2.08	7.05	8.95
γ‐GT	20.00	2.80	2.48	2.41	6.94	7.14
UA	17.00	3.87	2.15	1.93	6.11	6.80
ALP	30.00	6.99	3.98	3.32	5.78	6.93
AST	20.00	2.86	3.38	2.51	5.07	6.83
TC	10.00	1.11	2.15	2.11	4.13	4.21
TP	10.00	2.02	1.86	2.06	4.29	3.87
CRE	15.00	4.25	3.36	2.69	3.20	4.00
ALB	10.00	2.91	2.31	2.11	3.07	3.36
GLU	10.00	2.57	2.45	2.18	3.03	3.41
ALT	20.00	2.98	5.78	4.24	2.94	4.01
Ca	10.00	2.25	2.73	2.93	2.84	2.65
BUN	9.00	1.67	2.92	2.79	2.51	2.63
P	10.00	4.18	2.81	2.60	2.07	2.24
K	8.62	1.26	1.22	1.56	6.03	4.72
Na	7.40	1.20	1.24	1.31	5.00	4.73
Cl	5.00	1.50	1.48	1.55	2.36	2.26

**Table 2:** Sigma metrics (Levels 1 and 2) for 16 analytes obtained using the P2 analysis module of AU5800 calculated based on the TEa, bias (%), and CV (%, Levels 1 and 2)

Analytes	TEa (%, CLIA)	Bias (%)	CV (%)		Sigma	
			Level 1	Level 2	Level 1	Level 2
TG	25.00	2.00	2.28	2.40	10.09	9.58
CK	30.00	1.92	3.12	2.79	9.00	10.06
γ‐GT	20.00	2.80	2.00	1.82	8.60	9.45
TBIL	20.00	1.39	2.39	1.99	7.79	9.35
UA	17.00	3.87	1.77	1.88	7.42	6.98
ALP	30.00	6.99	3.67	2.65	6.27	8.68
AST	20.00	2.86	3.33	2.58	5.15	6.64
CRE	15.00	4.25	2.21	2.13	4.86	5.05
TP	10.00	2.02	1.71	1.56	4.67	5.12
TC	10.00	1.11	1.76	1.97	5.05	4.51
GLU	10.00	2.57	2.00	1.92	3.72	3.87
ALB	10.00	2.91	2.03	1.96	3.49	3.62
ALT	20.00	2.98	6.61	3.32	2.57	5.13
BUN	9.00	1.67	2.78	2.87	2.64	2.55
Ca	10.00	2.25	3.54	3.07	2.19	2.53
P	10.00	4.18	3.30	2.88	1.76	2.02

In the P1 and ISE modules, 11 of the 19 analytes showed a performance of at least 4σ (good) at QC material Level 1, and six of these analytes (CK, TG, TBIL, γ‐GT, UA, and K) presented world‐class performance (Table 1 and Figure 1). In addition, 12 of the 19 analytes showed a performance of at least 4σ (good) at QC material Level 2, and seven of these analytes (CK, TG, TBIL, γ‐GT, UA, ALP, and AST) presented world‐class performance (Table 1 and Figure 1). In the P2 module, 10 of 16 analytes showed a performance of at least 4σ (good) at QC material Level 1, and six of these analytes (TG, CK, γ‐GT, TBIL, UA, and ALP) presented world‐class performance (Table 2 and Figure 2). Moreover, 11 of 16 analytes showed a performance of at least 4σ (good) at QC material Level 2, and seven of these 11 analytes (TG, CK, γ‐GT, TBIL, UA, ALP, and AST) presented world‐class performance (Table 2 and Figure 2). The data demonstrated that the performance of five analytes (CK, TG, TBIL, γ‐GT, and UA) reached the Six Sigma level in both analysis modules and at both QC material levels and that nine analytes (TP, CRE, ALB, GLU, ALT, Ca, BUN, P, and Cl) exhibited σ < 4 at one or both QC material levels.

### 3.2 Quality control procedures selected by sigma metrics for the analytes

In the daily work of our clinical biochemical laboratory, the QC procedure empirically adopted involves the use of multi‐rules 1_2s_/2_2s_/1_3s_ with one control measurement at two QC material levels and running these rules once for all the analytes, with the exception of the out‐of‐control analytes. To investigate the appropriate QC procedures for these analytes with a high probability of error detection (*P*_ed_) and a low probability of false rejection (*P*_fr_), the concept of the statistical QC (SQC) procedure based on sigma metrics was introduced and adopted in this study according to the new guidance CLSI C24‐Ed4 18 and novel studies on SQC.^19^, ^20^ The design of the SQC procedure adopted in this study included the following three parameters: the selection of Westgard Sigma rules, the total number of control measurements per QC event (N), and the run size of patient samples between QC events (R). The QC procedures for the 19 analytes with different modules and at different QC material levels are detailed in Table 3. For example, if the analytes presented world‐class performance (σ ≥ 6), as observed for CK, TG, TBIL, γ‐GT, and UA, only one control rule, 1_3s_, with one control measurement at two QC material levels (N2) per QC event and a run size of 1000 patient samples between QC events (R1000), was needed for QC at both QC material levels and with both analysis modules (Table 3). However, if the analytes presented marginal, poor, or unacceptable performance (σ < 4) at one or both QC material levels, as observed for TP, CRE, ALB, GLU, ALT, Ca, BUN, P, and Cl, full multi‐rules, namely 1_3s_/2_2s_/R_4s_ /4_1s_/8_x_ with N4 and R45, would be adopted for QC (Table 3). For the other five analytes with 4 ≤ σ < 6 at one or both QC material levels, stricter rules, such as 2_2s_, R_4s_, and 4_1s_, were needed for QC, and higher N values and lower R values were accompanied by a decreased sigma grade. The data demonstrated that the sigma methodology can scientifically optimize the QC procedures for the analytes at every QC material level and with every module.

**Table 3:** QC procedures selected for 19 analytes

Analytes	P1 and ISE			P2		
	Sigma		QC procedure	Sigma		QC procedure
	Level 1	Level 2		Level 1	Level 2	
CK	9.30	9.96	1_3s_ with N2 and R1000	9.00	10.06	1_3s_ with N2 and R1000
TG	9.43	8.71	1_3s_ with N2 and R1000	10.09	9.58	1_3s_ with N2 and R1000
TBIL	7.05	8.95	1_3s_ with N2 and R1000	7.79	9.35	1_3s_ with N2 and R1000
γ‐GT	6.94	7.14	1_3s_ with N2 and R1000	8.60	9.45	1_3s_ with N2 and R1000
UA	6.11	6.80	1_3s_ with N2 and R1000	7.42	6.98	1_3s_ with N2 and R1000
ALP	5.78	6.93	1_3s_/2_2s_/R_4s_ with N2 and R450 (Level 1); 1_3s_ with N2 and R1000 (Level 2)	6.27	8.68	1_3s_ with N2 and R1000
AST	5.07	6.83	1_3s_/2_2s_/R_4s_ with N2 and R450 (Level 1); 1_3s_ with N2 and R1000 (Level 2)	5.15	6.64	1_3s_/2_2s_/R_4s_ with N2 and R450 (Level 1); 1_3s_ with N2 and R1000 (Level 2)
TC	4.13	4.21	1_3s_/2_2s_/R_4s_/4_1s_ with N4 and R200	5.05	4.51	1_3s_/2_2s_/R_4s_ with N2 and R450 (Level 1); 1_3s_/2_2s_/R_4s_/4_1s_ with N4 and R200 (Level 2)
TP	4.29	3.87	1_3s_/2_2s_/R_4s_/4_1s_ with N4 and R200 (Level 1); 1_3s_/2_2s_/R_4s_/4_1s_/8_x_ with N4 and R45 (Level 2)	4.67	5.12	1_3s_/2_2s_/R_4s_/4_1s_ with N4 and R200 (Level 1); 1_3s_/2_2s_/R_4s_ with N2 and R450 (Level 2)
CRE	3.20	4.00	1_3s_/2_2s_/R_4s_/4_1s_ /8_x_ with N4 and R45 (Level 1); 1_3s_/2_2s_/R_4s_/4_1s_ with N4 and R200 (Level 2)	4.86	5.05	1_3s_/2_2s_/R_4s_/4_1s_ with N4 and R200 (Level 1); 1_3s_/2_2s_/R_4s_ with N2 and R450 (Level 2)
ALB	3.07	3.36	1_3s_/2_2s_/R_4s_/4_1s_/8_x_ with N4 and R45	3.49	3.62	1_3s_/2_2s_/R_4s_/4_1s_/8_x_ with N4 and R45
GLU	3.03	3.41	1_3s_/2_2s_/R_4s_/4_1s_/8_x_ with N4 and R45	3.72	3.87	1_3s_/2_2s_/R_4s_/4_1s_/8_x_ with N4 and R45
ALT	2.94	4.01	1_3s_/2_2s_/R_4s_/4_1s_/8_x_ with N4 and R45 (Level 1); 1_3s_/2_2s_/R_4s_/4_1s_ with N4 and R200 (Level 2)	2.57	5.13	1_3s_/2_2s_/R_4s_/4_1s_/8_x_ with N4 and R45 (Level 1); 1_3s_/2_2s_/R_4s_ with N2 and R450 (Level 2)
Ca	2.84	2.65	1_3s_/2_2s_/R_4s_/4_1s_/8_x_ with N4 and R45	2.19	2.53	1_3s_/2_2s_/R_4s_/4_1s_/8_x_ with N4 and R45
BUN	2.51	2.63	1_3s_/2_2s_/R_4s_/4_1s_/8_x_ with N4 and R45	2.64	2.55	1_3s_/2_2s_/R_4s_/4_1s_/8_x_ with N4 and R45
P	2.07	2.24	1_3s_/2_2s_/R_4s_/4_1s_/8_x_ with N4 and R45	1.76	2.02	1_3s_/2_2s_/R_4s_/4_1s_/8_x_ with N4 and R45
K	6.03	4.72	1_3s_ with N2 and R1000 (Level 1); 1_3s_/2_2s_/R_4s_/4_1s_ with N4 and R200 (Level 2)	a	a	a
Na	5.00	4.73	1_3s_/2_2s_/R_4s_ with N2 and R450 (Level 1); 1_3s_/2_2s_/R_4s_/4_1s_ with N4 and R200 (Level 2)	a	a	a
Cl	2.36	2.26	1_3s_/2_2s_/R_4s_/4_1s_/8_x_ with N4 and R45	a	a	a

Note

N, total number of control measurements per run, N2 represents two measurements at a single control material level or one measurement at two control material levels, and a similar definition applies to N4; R, run size of patient samples between QC events, R1000 represents a run size of 1000 patient samples between QC events, and similar definitions apply to R450, R200, and R45.

a Not applicable.

### 3.3 Combination of QGI analysis and RCA for the analytes with σ < 4

According to the new guideline CLSI C24‐Ed4, the SQC procedures with *P*_ed_ ≥ 90% and *P*_fr_ ≤ 5% are recommended for analytes.^18^ However, nine analytes with σ < 4 under the SQC procedure consisting of the full multi‐rules 1_3s_/2_2s_/R_4s_/4_1s_/8_x_ with N4 and R45 could not meet this requirement. Thus, to assure quality and determine why these analytes did not reach the 4σ level or above, the QGI ratios were identified. Five analytes (TP, ALT, Ca, BUN, and Cl) showed imprecision problems at one or more QC material levels; three analytes (CRE, ALB, and P) exhibited both inaccuracy and imprecision problems at one or more QC material levels; and the remaining analyte (GLU) showed low precision with the P1 module and both accuracy and precision problems with the P2 module (Table 4).

**Table 4:** QGI analysis for analytes with σ < 4

Analyte	P1 and ISE		P2		Problem
	Level 1	Level 2	Level 1	Level 2	
TP	a	0.65	a	a	Imprecision
CRE	0.84	a	a	a	Imprecision and inaccuracy
ALB	0.84	0.92	0.96	0.99	Imprecision and inaccuracy
GLU	0.70	0.79	0.86	0.89	Imprecision (P1) Imprecision and Inaccuracy (P2)
ALT	0.34	a	0.30	a	Imprecision
Ca	0.55	0.51	0.42	0.49	Imprecision
BUN	0.38	0.40	0.40	0.39	Imprecision
P	0.99	1.07	0.84	0.97	Imprecision and inaccuracy
Cl	0.68	0.65	a	a	Imprecision

^a^ Not applicable.

**Figure 3:**
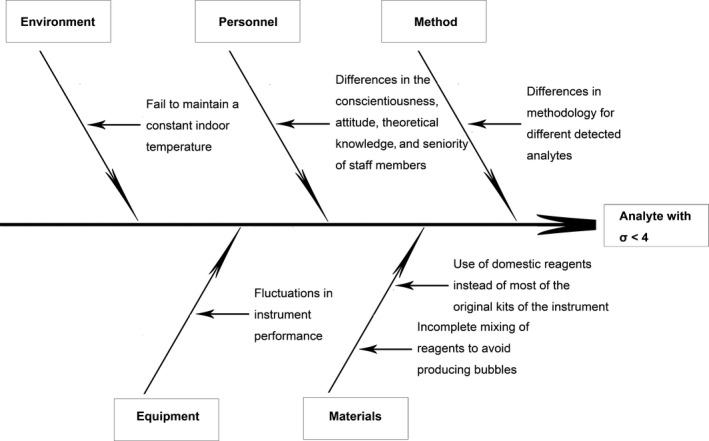
Cause‐effect chart (fishbone diagram) of potential causes for analytes with σ < 4 at one or more QC levels

To further detect the root causes of the problems with these analytes, a cause‐effect chart was used as a technical tool for RCA. As shown in Figure 3, five aspects of potential root causes, including aspects related to methodology, materials, personnel, equipment, and working conditions, were investigated. For example, to analyze the methodology and personnel factors, 14 analytes (nine analytes with σ < 4 and five analytes with world‐class performance) investigated by six staff members were evaluated based on sigma metrics (Table S1). The same staff members worked under the same conditions using the same QC material level, the same domestic brand of reagents (with the exception of the electrolytes using the original reagents), and the same module. As a result, five analytes (CK, TG, TBIL, γ‐GT, and UA) with world‐class performance could generally reach at least the 5σ level, whereas P exhibited a performance of σ < 4 regardless of the personnel factor, as shown in Table S1. This finding demonstrated that the performance of these analytes showed differences related to the methodology, which revealed that some methods were favorable and others were not appropriate. Therefore, reevaluating and improving the methodology used for the analytes would improve the quality. In addition, the performance of the same analyte obtained with different staff members presented different sigma levels, as observed with TP, CRE, ALB, GLU, ALT, Ca, BUN, P, and Cl (Table S1). The potential reason for this finding might be that staff members exhibit different degrees of conscientiousness, attitude, theoretical knowledge, and seniority, which demonstrated that the personnel factor plays a role in the performance of the analytes. Thus, personnel retraining as well as a review of the standard operating procedures (particularly those used for reagent addition) and a reevaluation of the competency of some staff members might be favorable for improving quality.

Together, combining imprecision or inaccuracy problems with the potential five aspects of root causes might constitute a strategy for solving problems related to these nine analytes and improving their quality.

## 4 DISCUSSION

**Figure 4:**
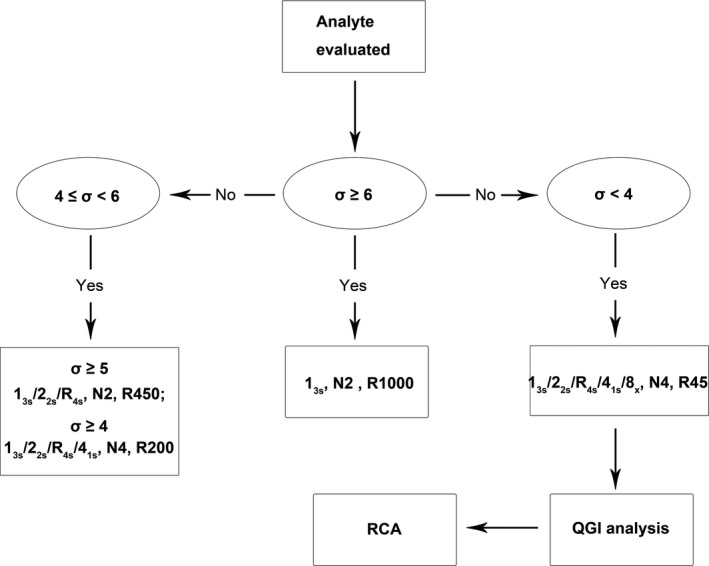
Process map for the Six Sigma management workflow

In this study, we analyzed 19 biochemical analytes using sigma methodology. The Six Sigma management workflow for quality assurance and improvement is summarized in Figure 4. First, each analyte was effectively evaluated according to the sigma value. Second, the QC procedures were optimized and individualized for the analytes with different sigma grades. Third, the detected QGI ratios and RCA further revealed that the accuracy or precision of the analytes with performance below the 4σ level needed to be improved and revealed five aspects of potential root causes.

In clinical settings, the credibility of clinical reports relies on two items: precision and accuracy. Sigma metrics reveal errors or defects in precision and accuracy that can be used to evaluate quantitative projects. Thus, the Six Sigma methodology was evaluated in our work. Surprisingly, the sigma levels of a few of the analytes investigated in this study, such as TP, BUN, and GLU, showed variations among different research groups.^3^, ^6^, ^10^ This phenomenon could be attributed to two points: One was the detection system, including the different types of analyzers, reagents, and QC materials used, as well as other pre‐analytical and analytical conditions; and the other was the source selection of the TEa targets and the slight differences in the algorithms used to evaluate the bias and CV, which might affect the sigma values. TEa targets selected to meet our laboratory's requirement and reasonable algorithms for calculating bias and CV values were used in this study to calculate the sigma values for every analyte. Although the accumulated bias value was adopted in this study, the impact on the sigma calculation was slight because the bias values for these analytes were relatively stable according to the long‐term EQA results. More interestingly, wide variations in sigma values between the two QC material levels and the two analysis modules were discovered for ALP, CRE, TP, ALT, and ECT in this study (Tables 1 and 2). However, this situation was not particular to this study because it was also discovered in other studies.^6^, ^7^, ^8^, ^9^, ^10^, ^11^ The two analysis modules could be considered two separate analyzers, and differences in performance could not be avoidable. However, the sigma levels obtained with these two analysis modules were generally comparable. The discrepancy between the two material levels was partly attributed to the methodology used for some analytes, which might present better performance with normal or abnormal concentrations of the QC materials. Thus, as suggested by a previous study, stricter QC procedures should be followed under these conditions to abolish this discrepancy.^11^ Various corrective actions were performed in this study for these analytes, as shown in Table 3.

The IQC procedure is an important stage in the daily work performed in clinical settings. As previously reported, appropriate QC procedures might not only decrease the *P*_fr_ and increase the *P*_ed_ but also avoid economic costs and improve efficiency.^21^, ^22^ For example, compared with the previous procedures adopted in our laboratory, only one QC rule, 1_3s_, needed to be used for TG, CK, γ‐GT, TBIL, and UA, which decreased economic costs and increased the working efficiency. However, for the analytes with σ < 6, more rigorous QC procedures were implemented in this study compared with those used previously because the quality of clinical results and benefits to patients are of primary importance as long as the associated costs are reasonable. However, there remain two problems associated with the practical implementation of the QC procedures as recommend by the current study: To address the fact that different QC procedures are needed for some analytes (such as ALP, AST, and TC) at the two QC material levels, stricter QC procedures should be implemented uniformly rather separately; the QC procedures for analytes with σ < 4 are quite unpractical because 26 runs of QC materials at two QC materials levels are needed for the 600 patient samples that are investigated each day in our laboratory. Thus, improving the quality of these nine analytes was a major problem that needed to be addressed.

To improve the quality of these analytes, a strategy that combines QGI analysis with RCA for problem discovery was proposed in this study. QGI provided robust directions for solving only the problems associated with the analytes, such as inaccuracy or imprecision. However, the shortcomings of the QGI analysis could be compensated with RCA. In the analytical process, the observed problems belonged to five factors, as shown in Figure 3. Of course, the potential root causes included in the figure are only based on the situation in our laboratory, and other undiscovered problems might also exist. As shown in a previous study in a veterinary laboratory, methodology improvements (reagent substitution) and personnel training can improve the quality of analytes.^23^ Therefore, addressing the method and personnel factors could improve the quality of some analytes with low sigma values, such as P and Cl. In addition, quality problems remained due to failures at multiple levels of the measurement processes, indicating the existence of multiple root causes, which is consistent with the adverse events observed in health care.^15^ The problems associated with working conditions and instrument proficiency could also affect measurement quality, and these problems cannot be ignored (Figure 3). For example, the analyzer sometimes emits a high‐temperature alarm once in summer, which is inevitably linked to the environmental temperature due to the lack of a constant indoor temperature. This situation would impact not only the instrument proficiency but also the enzymatic methods used for the analytes. Thus, designing a constant‐temperature system for use in a laboratory would help resolve this problem. To address fluctuations in instrument proficiency and thus improve quality, the frequency of calibrating these analytes could be increased from once a week to every 2 days in our laboratory. The degree of improvement in the quality of these analytes will be investigated in our future work. Certainly, if the performance of an analyte cannot be improved by implementation of all the proposed actions, nonstatistical QC procedures, including repeated tests for a patient and comparability testing, could be adopted for QA, as suggested by previous studies.^18^, ^24^

Overall, the Six Sigma methodology provides a useful evaluation system for the biochemical projects considered in this study, optimizes the QC procedures for every item, and supplies a problem‐solving strategy for analytes with σ < 4. This method has great practical value in clinical biochemical laboratories.

## AUTHOR CONTRIBUTIONS

BZ, YC, and YW conceived and designed the approach of this study; HH collected the 5‐month daily quality data; BZ analyzed data and wrote the article; and YC, CL, and LT supervised this study and reviewed and edited this article. All authors have read and approved the final manuscript.
