# Supplementary material for: Practical application of Six Sigma management in analytical biochemistry processes in clinical settings
Source: J Clin Lab Anal. 2019 Nov 27;34(1):e23126. doi: 10.1002/jcla.23126 (PMC6977137; doi:10.1002/jcla.23126)
Supplement: Supplementary file 1 [file JCLA-34-e23126-s001.doc]

Supplementary Table 1 The performance of 14 analytes evaluated with sigma metrics (Levels 1/2 and P1/2) by six staff members.

| Analyte | A (19d) | | | | B (33d) | | | | C (8d) | | | | D (8d) | | | | E (11d) | | | | F (11d) | | | |
| --- | --- | --- | --- | --- | --- | --- | --- | --- | --- | --- | --- | --- | --- | --- | --- | --- | --- | --- | --- | --- | --- | --- | --- | --- |
|  | P1 | | P2 | | P1 | | P2 | | P1 | | P2 | | P1 | | P2 | | P1 | | P2 | | P1 | | P2 | |
|  | L1 | L2 | L1 | L2 | L1 | L2 | L1 | L2 | L1 | L2 | L1 | L2 | L1 | L2 | L1 | L2 | L1 | L2 | L1 | L2 | L1 | L2 | L1 | L2 |
| TP | † | 3.26 | † | † | † | 3.93 | † | † | † | 7.96 | † | † | † | 2.79 | † | † | † | 4.30 | † | † | † | 6.08 | † | † |
| CRE | 4.20 | † | † | † | 3.08 | † | † | † | 2.92 | † | † | † | 3.04 | † | † | † | 3.29 | † | † | † | 3.59 | † | † | † |
| ALB | 3.16 | 3.52 | 5.37 | 4.75 | 3.45 | 3.23 | 3.29 | 3.17 | 6.40 | 5.23 | 6.03 | 9.70 | 2.63 | 3.24 | 3.96 | 6.45 | 2.88 | 3.37 | 2.88 | 2.67 | 3.74 | 4.84 | 4.75 | 5.80 |
| GLU | 3.54 | 3.30 | 3.38 | 4.28 | 3.26 | 3.76 | 4.12 | 4.11 | 6.03 | 3.76 | 9.35 | 3.53 | 4.10 | 3.99 | 6.30 | 3.94 | 2.66 | 3.27 | 2.61 | 3.90 | 4.11 | 5.08 | 5.69 | 3.99 |
| ALT | 2.84 | † | 2.38 | † | 2.87 | † | 2.34 | † | 4.77 | † | 2.34 | † | 1.95 | † | 2.78 | † | 3.43 | † | 2.33 | † | 3.51 | † | 3.36 | † |
| BUN | 2.45 | 1.98 | 2.38 | 2.02 | 2.73 | 3.14 | 2.86 | 3.36 | 4.04 | 6.27 | 2.60 | 2.97 | 1.99 | 1.80 | 2.10 | 1.95 | 2.10 | 3.06 | 3.93 | 3.50 | 2.55 | 2.33 | 3.09 | 2.55 |
| Ca | 2.77 | 1.96 | 4.04 | 4.80 | 2.80 | 2.69 | 1.37 | 1.54 | 5.51 | 4.37 | 2.94 | 3.65 | 2.28 | 2.04 | 4.27 | 4.18 | 2.97 | 3.24 | 2.84 | 3.87 | 3.39 | 4.73 | 6.65 | 6.22 |
| P | 1.80 | 1.81 | 1.90 | 1.93 | 2.74 | 2.54 | 1.92 | 2.24 | 1.97 | 3.45 | 2.57 | 3.15 | 1.65 | 2.08 | 1.32 | 1.76 | 2.19 | 2.10 | 1.78 | 1.76 | 3.62 | 2.58 | 2.11 | 2.33 |
| Cl | 2.83 | 2.36 | † | † | 3.20 | 2.88 | † | † | 3.24 | 3.48 | † | † | 2.37 | 1.83 | † | † | 2.49 | 1.94 | † | † | 1.50 | 1.96 | † | † |
| CK | 10.63 | 12.89 | 9.44 | 12.95 | 8.51 | 10.58 | 8.86 | 9.94 | 8.92 | 8.36 | 8.33 | 8.64 | 10.74 | 13.01 | 7.06 | 9.06 | 11.36 | 13.42 | 9.61 | 8.97 | 8.41 | 9.83 | 9.58 | 7.87 |
| TG | 14.12 | 7.58 | 10.93 | 10.79 | 9.22 | 8.54 | 9.51 | 11.02 | 14.07 | 13.84 | 12.10 | 13.91 | 8.07 | 10.95 | 10.57 | 14.98 | 8.25 | 8.53 | 13.61 | 10.34 | 11.56 | 8.18 | 14.04 | 20.02 |
| γ-GT | 9.06 | 8.02 | 7.28 | 9.29 | 5.80 | 6.45 | 8.13 | 10.14 | 8.95 | 11.20 | 19.87 | 14.31 | 5.78 | 6.85 | 6.07 | 7.01 | 9.11 | 9.87 | 11.19 | 9.39 | 12.80 | 11.95 | 8.83 | 14.13 |
| TBIL | 5.71 | 6.21 | 6.07 | 7.21 | 6.64 | 10.71 | 8.30 | 11.04 | 5.40 | 16.86 | 9.46 | 18.10 | 7.22 | 10.58 | 10.80 | 10.89 | 11.73 | 10.13 | 13.52 | 13.76 | 18.43 | 11.82 | 10.72 | 12.78 |
| UA | 6.43 | 8.19 | 7.05 | 7.77 | 6.03 | 7.41 | 8.94 | 9.54 | 6.46 | 5.50 | 19.28 | 17.60 | 10.47 | 6.18 | 5.05 | 5.64 | 6.88 | 9.68 | 8.21 | 5.46 | 7.82 | 9.98 | 9.60 | 9.25 |

Footnotes:

1. †, not applicable; d, the total number of days for IQC; L1, Level 1; L2, Level 2.
2. Staff A, 35-year seniority, technologist;

Staff B, 18-year seniority, associate senior technologist;

Staff C, 25-year seniority, associate senior technologist;

Staff D, 12-year seniority, associate senior technologist;

Staff E, 1-year seniority, technologist;

Staff F, 18-year seniority, full senior technologist.
